# Case Report: Aarskog-scott syndrome caused by FGD1 gene variation: A family study

**DOI:** 10.3389/fgene.2022.932073

**Published:** 2022-08-16

**Authors:** Yijia Liang, Honglin Wu, Xiumei He, Xiyu He

**Affiliations:** ^1^ Fifth Medical Center of Chinese PLA General Hospital, Beijing, China; ^2^ Chinese PLA Medical College, Beijing, China

**Keywords:** aarskog-scott syndrome, rare syndromes, FGD1 gene, family study, brother contact

## Abstract

Aarskog-Scott syndrome is a rare genetic disorder characterized by short stature, abnormal facial features, and digital and genital deformities. FGD1 gene variation is the known cause of this disorder. This paper described a Chinese family study of Aarskog-Scott syndrome in which the main patients were two brothers. Then, the relationship between genotype and phenotype in Aarskog-Scott syndrome was investigated preliminarily. A new FGD1 gene variant was revealed in this study, providing insights into the link between phenotype and genotype variations in Aarskog-Scott syndrome as well as a foundation for its diagnosis and treatment.

## 1 Background

Aarskog-Scott syndrome (AAS, MIM number 305400), also known as facio-digital-genital dysplasia, was first described by Aarskog in 1970 (Aarskog, 1971) and later detailed by Scott (Scott and Moyer, 1971). AAS is a rare genetic disorder characterized by short stature, abnormal facial features, and digital and genital deformities. Although FGD1 gene variation is currently the known genetic cause of this disorder and its pathophysiology has been elucidated, any other causes that might be involved need to be yet explored. AAS is an X-linked recessive inherited disease with strong genetic diversity. Aarskog-Scott syndrome most commonly occurs in Europe, the United States, Japan, South Africa, and India, but in China, only a few cases have been reported. Herein, we aimed to present a unique occurrence of Aarskog-Scott syndrome in a Chinese family and analyze the relationship between genotype and phenotype of this disorder. Further, we planned to include relevant literature review.

## 2 Clinical information

**FIGURE 1:**
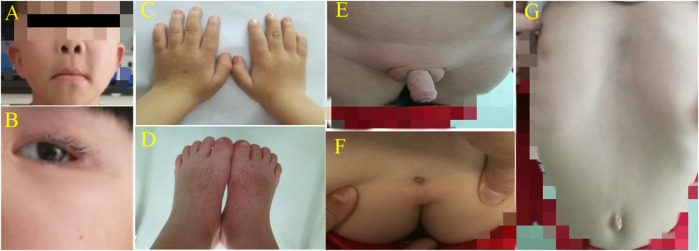
**(A)** + **(B)** High eyebrow arch, wide eye distance, small eye fissure, double eyelid ptosis, flat nose bridge and slightly upturned nostrils; the middle sulcus was wide and long, and the ear was low and backward. **(C)** + **(D)** Hands/soles were small; fingers/toes were short and thick; little fingers on both hands were short and curved with a single finger fold. **(E)** Bilateral oblique inguinal hernia and shapeless scrotum complicated with cryptorchidism (after surgery). **(F)** Sacral tail skin depression. **(G)** Sternum depression, costal margin eversion and a flat navel.

**FIGURE 2:**
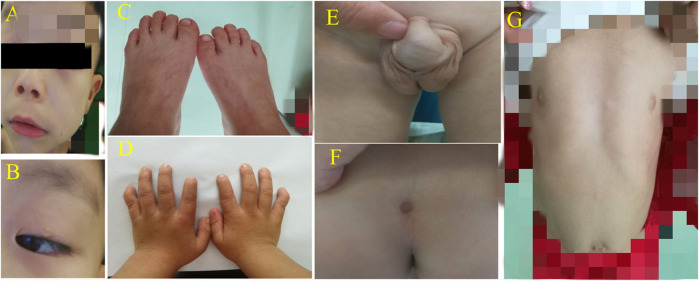
**(A)** + **(B)** High eyebrow arch, widening eye distance, small eye fissure and double eyelid ptosis; the nose bridge was flat, the nostrils were slightly elevated, the middle sulcus was wide and long, and the ear was low and backward. **(C)** + **(D)** Small hands/soles, short fingers/toes, and short little fingers curved with a single finger fold. **(E)** Shapeless scrotum. **(F)** Sacral caudal skin depression. **(G)** Sternal depression, outside costal margin turn and a flat navel.

The patients were two brothers. The elder brother was 7 years and 11 months old, and the younger one was 4 years and 9 months old. The cases were admitted to our hospital with the complaints of short stature and multiple deformities, they were born with full-term delivery, and their parents are non-consanguineous. The family reported that the growth retardation began at the age of one, and the birth weight and length were unknown. Nevertheless, no special attention was paid to these abnormalities and no intervention was performed. The antenatal history, birth event, and perinatal history were unremarkable. But specific growth and developmental milestones were overlooked. At admission to the hospital, the older boy’s height and weight were 109.3 cm (−3.7 SD) and 21.5 kg (−1.26 SD) respectively, while the younger boy’s height and weight were 100.4 cm (−1.3 SD) and 14.6 kg (−1.3 SD) respectively. Upon physical examination, older boy had a short and uniform body, small hands and soles, short and thick fingers and toes, short and curved little fingers on both hands with single finger pleats, high eyebrow arch, wide eye distance, wide eye cleft, drooping double eyelids, amblyopia, low and flat nose bridge, slightly upturned nostrils, low back and flapping ears, wide and long middle groove, sternal depression, oblique costal margin, inguinal hernia, shapeless scrotum with cryptorchidism (postoperatively), and sacral tail skin depression (Figure 1). The younger boy exhibited a proportionate short stature, small hands and soles, short and thick fingers and toes, short and curved little fingers on both hands with a single finger fold, wide eye distance and small eye cleft, double eyelid drooping, low nose bridge, slightly upturned nostrils, low and backward ear position, wind flapping ear, costal margin evagination, flat navel, shapeless scrotum, and sacral tail skin depression (Figure 2). Furthermore, the older boy had a long response time, hyperactive response, and inability to concentrate.

The father was 172 cm and the mother is 160 cm in height. The mother had bent little fingers both sides. Their elder sister displayed slight deformities. The patients’ cousin, three of their aunts and grandma had bent little fingers. The parents said that their late uncle’s height was normal, with facial micro-deformity (specific complaint was unspecified), short and thick digits, and bent little fingers both sides.

Through a battery of laboratory tests on both boys, the older boy had normal liver and kidney functions, as well as normal blood routine parameters and electrolyte levels. Regarding thyroid function, T3:1.28 nmol/L (1.02–2.96 nmol/L), T4: 80.20 nmol/L (55.5–161.3 nmol/L), FT3:6.1 pmol/L (2.8–6.3 pmol/L), FT4: 20.01 pmol/L (11.5–22.7 pmol/L), TSH: 2.31 µIU/ml (0.64–6.27 µIU/L). IGF-1:441 ng/ml (111–551 ng/ml). IGFBP-3: 6.83 µg/mL (2.7–8.9 µg/mL). HbA1c: 4.5% (4.8–5.9%). Fasting insulin:8.12 µU/mL (2.60–24.90 µU/mL). ACTH:18.35 pg/ml (10–52 pg/ml), Cortisol:6.63 µg/dL (2.5–20 µg/dL).17-αhydroxyprogesterone:0.249 ng/ml (0.07–3.6 ng/ml). The peak of growth hormone exercise screening test:5.167 ng/ml. Echocardiography, abdominal echocardiography and reproductive echocardiography were normal. Bone age assessed by imaging was about 6 years old, and pituitary magnetic resonance examination displayed no abnormalities. Karyotype analysis revealed 46, XY. And the younger boy had normal liver and kidney functions, as well as normal blood routine parameters and electrolyte levels. Regarding thyroid function, T3:2.10 nmol/L (1.02–2.96 nmol/L), T4: 105.4 nmol/L (55.5–161.3 nmol/L), FT3:3.3 pmol/L (2.8–6.3 pmol/L), FT4:13.5 pmol/L (11.5–22.7 pmol/L), TSH:2.66 µIU/ml (0.64–6.27 µIU/L). IGF-1:127 ng/ml (50–286 ng/ml). IGFBP-3: 4.37 µg/mL (1.8–6.4 µg/mL). HbA1c: 4.5% (4.8–5.9%). Fastinginsulin:7.34 µU/mL (2.60–24.90 µU/mL). ACTH:16.89 pg/ml (10–52 pg/ml), Cortisol:5.34 µg/dL (2.5–20 µg/dL).17-αhydroxyprogesterone:0.179 ng/ml (0.07–3.6 ng/ml). The peak of growth hormone exercise screening test:3.770 ng/ml. Echocardiography, abdominal echocardiography and reproductive echocardiography were normal. Bone age assessed by imaging was about 6 years old, and pituitary magnetic resonance examination displayed no abnormalities. The karyotype was 46, XY.

The trio whole-exome sequencing and Sanger sequencing were performed after informed consent was obtained from parents. Briefly, 2 ml peripheral blood was collected using blood collection tubes coated with EDTA anticoagulant, from the two subjects. Genomic DNA was extracted from peripheral blood using a genomic DNA extraction kit (Kangwei Century Biotechnology Co., Ltd., Beijing, China) and the DNA quality was above the grade D. DNA was fragmented (10–700 bp) by ultrasonic apparatus (Covaris S220 ultrasonicator; Covaris, Woburn, MA, United States). PCR machine was used to amplify the DNA fragments and magnetic beads (Ampure beads: PCR products was 1.5:1) were used to purify the PCR amplicons. Standard library construction kit (independently developed by Mackinac) was used to construct DNA library, and the target region of the library was captured and quantified using GenCap liquid phase capture kit (independently developed by Mackinac) and KAPA qPCR kit (Roche Diagnostics Corporation, Indianapolis, IN, United States) on a PCR instrument. Finally, the high-throughput sequencing system Illumina NextSeq 500 was used for sequencing. After pre-processing and analysis of relevant data, pathogenicity analysis was performed following the American College of Medical Genetics and Genomics (ACMG)guidelines.

The results showed that a *FGD1* gene hemizygous variation was present in both cases, which was derived from their mother and GRCh37/HG19 was used as the reference genome. The *FGD1* gene, located on chrX- 54481880, was screened for variations, and the c.2015 + 1G>A variation was detected in exon 12 (RefSeq: NM_004,463). As a result, nucleotide no. 2015 + 1 in the coding region was changed from guanine to adenine. This was therefore a splicing variation. According to the ACMG guideline, this variant is pathogenic. However, this variant has not yet been reported in Clinvar database and HGMD professional edition database.

In conclusion, both cases were clinically diagnosed as Aarskog-Scott syndrome. Since the patients did not want to do other kinds of growth hormone stimulation experiments and the peak value of the pervious experiment was low, we directly treated the children with growth hormone with the consent of their parents.

Both patients were treated with long-term follow-up. Both boys were given with long-acting growth hormone at 0.2 mg/kg/w on a regular basis with the informed consent from parents. Until now, both cases have been on regular medication for more than 4 years. Throughout the time, periodic monitoring was done on both patients, including fasting blood glucose level, fasting insulin level, glycosylated hemoglobin content, and bone age evaluation. After 1 year, the height and weight of the older boy were 118 cm (−3.00 SD) and 25.6 kg (−0.86 SD) while the height and weight of the younger boy were 111 cm (−0.82 SD) and 20.3 kg (0 SD), respectively, suggesting that the children had catch-up growth in height and weight, which was basically consistent with the research results of Feyza et al. (Darendeliler et al., 2003). At the last follow-up, the height and weight of the older boy were 144 cm (−1.31 SD) and 45 kg (−0.14 SD) while the height and weight of the younger boy were 135 cm (0 SD) and 27 kg (−0.55 SD), respectively.

**FIGURE 3:**
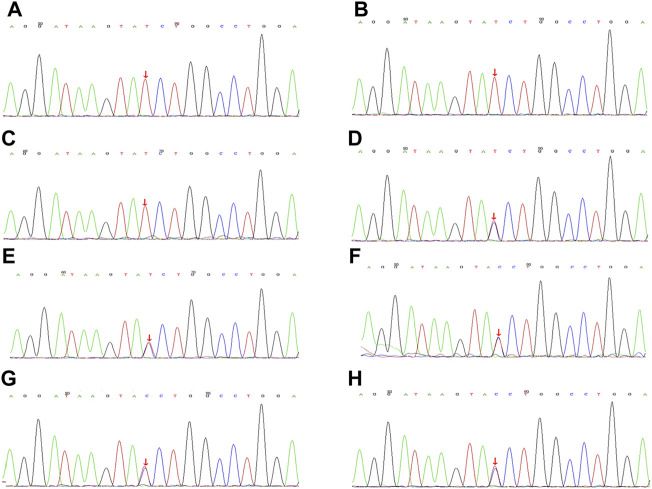
Gene sequencing of family members with Aarskog-Scott syndrome, with the variation shown by arrows. **(A)**. Case 1, FGD1 gene with **(C)**.2015 + 1G>A semi-heterozygous variation. **(B)**. Case 2, FGD1 gene with **(C)**.2015 + 1G>A semi-heterozygous variation. **(C)**. The children’s cousin FGD1 gene with **(C)**.2015 + 1G>A semi-heterozygous variation. **(D)**. FGD1 gene with **(C)**.2015 + 1G>A heterozygous variation. **(E)** FGD1 gene with **(C)**.2015 + 1G>A heterozygous variation. **(F)**. FGD1 gene with **(C)**.2015 + 1G>A heterozygous variation. **(G)** FGD1 gene with **(C)**.2015 + 1G>A heterozygous variation. **(H)**. FGD1 gene with **(C)**.2015 + 1G>A heterozygous variation.

**FIGURE 4:**
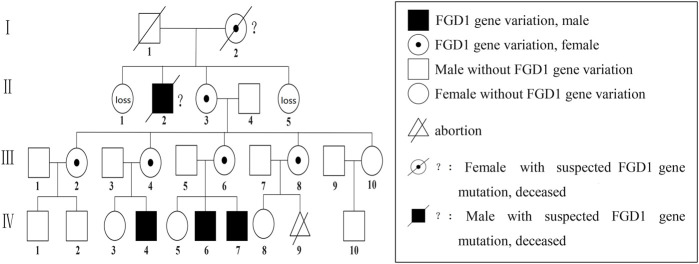
Aarskog-Scott syndrome family diagram; Ⅳ-4,Ⅳ-6, and Ⅳ-7 had FGD1 gene C.2015 + 1G>A semi-heterozygous variation, including Ⅳ-6 and Ⅳ-7 who were our patients. C.2015 + 1G>A heterozygous variation of the FGD1 gene for Ⅲ-2, Ⅲ-4, Ⅲ-6, and Ⅲ-8; Ⅰ-2 andⅠ-2 had died, and were speculated to have harbored FGD1 gene variation.

The presence of *FGD1* gene variation in the family was suspected based on the family history of cases and the nature of X-linked recessive inheritance of *FGD1* gene variation in Aarskog-Scott syndrome. The surviving members of the family were subjected to the genetic tests. The results are shown in Figure 3 and the family diagram is depicted in Figure 4.

Aarskog-Scott syndrome was considered due to the inheritance nature based on clinical reports and genetic test results. We described the clinical phenotypes of two patients with Aarskog-Scott syndrome in this lineage and identified a new FGD1 gene variant that broadened the spectrum of FGD1 variations causing Aarskog-Scott syndrome. These findings provided a foundation for the diagnosis and correlation between phenotype and genotype in Aarskog-Scott syndrome.

## 3 Discussion

AAS is a rare disease with genetic heterogeneity, and X-linked recessive inheritance is the most common inheritance mode (Escobar and Weaver, 1978) (Grier et al., 1983); FGD1 gene variation is the only confirmed etiological factor for AAS.

The role of *FGD1* gene variants in the pathogenesis of X-linked AAS was first described in Germany based on a familial translocation breakpoint analysis (Pasteris et al., 1994). The FGD1 gene, located at Xp11.22, about 51 kb in length, contains 18 exons, and encodes a protein containing 961 amino acids, namely guanine nucleotide conversion factor (GEF), which contains five conserved domains (proline rich domain, DH domain, FYVE domain, and 2 PH domains) (Orrico et al., 2000). GEF encoded by the FGDI gene specifically activates the Rho GTPase CDC42, regulating actin morphology and protein kinase/C-Jun N-terminal kinase to control cell proliferation and differentiation through DH and PH domains that catalyze THE release of GDP, as well as binding of GTP. By altering bone development and morphogenesis and regulating extracellular matrix accumulation, GEF acts on the whole-body skeleton, superficial facial structures, eyes, genitals, and nervous system to maintain the normal morphology, development and function of corresponding tissues and organs (Pasteris et al., 1997). Variations in the FGD1 gene lead to the production of dysfunctional proteins that interfere with the CDC42 signaling, resulting in generalized developmental abnormalities. After knocking out the mutant MLK3 allele that is resistant to FGD1/CDC42 activation, mice showed similar skeletal defects, suggesting that FGD1/CDC42-activated MLK3 was critical for bone mineralization (Zou et al., 2011), confirming the influence of FGD1 gene variation on bone development.

FGD1 was also reported to cause cortical malformations through the CDC42 pathway, resulting in attention deficits and impaired intelligence. Fryns et al. showed that at least 30% of patients with AAS displayed intellectual disability, including mild intellectual disability in two-third of the 30% (Fryns, 1992). However, a study on 456 cases in Qinba Mountains with FGD1 gene polymorphisms and intellectual development disorders (IDD) showed that IDD was not significantly associated with the gene frequency, genotype frequency, and haplotype of the five SNP FGD1 loci, suggesting that FGD1 may have not been the single X-linked factor (Li et al., 2014). The specific mechanism by which FGD1 gene/CDC42 pathway induces brain dysplasia and intellectual disability remains to be further explored. The expression of the CDC42 protein was decreased in Xp11, which was predisposed to schizophrenia. A study on four unrelated Chinese male XP11.22 duplicative patients mainly with intellectual disability, language impairment, and motor delay speculated that FGD1 may have been the potential dose-sensitive gene associated with hypogonadism in these patients (Wang et al., 2020). In addition, FGD1 was confirmed as a transforming growth factor-β (TGF-β), regulating guanine nucleotide conversion factor (GEF) that was involved in cytokine-induced nuclear liposome formation, which may have been part of the molecular basis of vascular lesions (Daubon et al., 2011).

The phenotype of Aarskog-Scott syndrome (AAS) is varied. Short stature associated with Aarskog has craniofacial abnormalities such as hyperthyroidism, short nose, ptosis, and genital deformities such as cape scrotum and shawl cryptorchidism (Aarskog, 1971). Scott, on the other hand, described the same features in three different patients (Scott and Moyer, 1971). As time goes on, several other authors reported similar cases, describing the phenotype of these patients as characterized by the presence of various associated signs such as oblique, short, long middle, widow’s peak, Camptodactyly, interphalanx webbed, and inguinal/umbilical hernia (P´erez-Coria et al., 2015) (Niida et al., 2014).

Teebi et al. established clear diagnostic criteria by summarizing the features of reported AAS in 1993. Main diagnostic symptoms included short stature, abnormal facial features, short nose with forward slope, maxillary hypoplasia, lower lip wrinkle, mildly crossed fingers, wide and short palms, short and curved little fingers, and shapeless scrotum. Secondary clinical diagnostic symptoms were thick external ear with excessive backward slant, forehead “V" tip, ptosis, downward slant palpebral fissure, hyperextension of joints, wide feet with clubbed toes, inguinal hernia, hypospadias, and navel abnormality. In clinical practice, due to the superposition of clinical phenotypes, it is often necessary to distinguish AAS from other genetic diseases such as Robin syndrome, Optiz syndrome, Bloom syndrome, Noonan syndrome, and pseudo-parathyroidism. Recent studies showed that patients with AAS showed multiple abnormalities, including anatomic abnormalities such as cardiovascular system symptoms (e.g., aortic stenosis and right ventricular hypertrophy) (Fernandez et al., 1994), nerve mental symptoms such as bipolar disorder, ADHD, autism spectrum disorders, epilepsy, Asperger’s syndrome, and intellectual disability (Nayak et al., 2012), and musculoskeletal system conditions such as symmetrical distal joint disease, myopathy, hip dislocation, finger and knee jerk before stretching, and flat feet (Zielinski and Pack, 2008), Moreover, Bayat and Calabrese both reported that myopathic involvement should be considered in AAS (Bayat et al., 2022) (Calabrese et al., 2022).

The manifestations of both cases in this family were in line with the main diagnostic indicators mentioned above such as uniformly short stature, small hands and soles, short and thick fingers and toes, short and curved little fingers on both hands with a single finger fold, wide eye distance and small eye cleft, low and flat nose bridge, slightly upturned nostrils, and shawl like scrotum. Secondary indicators such as double eyelid drooping, low and back ear position, and flat navel were also aligned with the stated symptoms. Of note, the older brother had significant neurological symptoms such as hyperactivity, attention deficit, and intellectual disability, but the younger brother did not. The clinical phenotypes of both cases were summarized as short stature, abnormal facial features, and digital and genital deformities. But the elder brother experienced with slight neurological abnormalities, which was in line with current understanding of abnormal phenotypes in AAS. Further, both patients had semi-heterozygous FGD1 gene variation, which provided strong evidence for the association between FGD1 gene variation and AAS phenotype. By analyzing seven individuals with FGD1 gene variation in three generations of this family, it was considered that AAS displayed the typical X-linked recessive inheritance pattern in this family. There was a FGD1 gene variation in the X chromosome of the grandmother of the children, which was transmitted to their mother and three aunts. Then, the mutated gene was transmitted from mother to both boys. Due to the obvious gender specificity of AAS (Balaton et al., 2018), both boys had typical clinical features of AAS, while their mother, aunt and grandmother only showed slight deformities in fingers.

Drumond et al. conducted a systematic analysis of the correlation between genotype and phenotype in AAS patients, but found no causal relationship between the two (Zanetti et al., 2021). Comparative analysis showed that our patients met the clinical diagnostic criteria of AAS and had a new *FGD1* gene variant, but no significant genotype-phenotype relation was found in the family members.

Recently, a study on AAS syndrome in an Italian family reported the variant C.1828C>T (p. Arg610*) in the FGD1 gene with 16P microdeletion, a genetic variant, but still with unclear phenotypic consequences, which indicated a potential association between AAS and chromosomal disorders (Pavone et al., 2020).

Presently, no studies have clearly confirmed the association between FGD1 genotype and phenotype in Aarskog-Scott syndrome. In the future, more cases combined with new gene testing methods could be used to elucidate the pathological mechanism causing specific genotype and phenotype in Aarskog-Scott syndrome to identify effective intervention measures for this syndrome.

## Data Availability

The datasets for this article are not publicly available due to concerns regarding participant/patient anonymity. Requests to access the datasets should be directed to the corresponding author.
